# Differential Induction of Functional IgG Using the *Plasmodium falciparum* Placental Malaria Vaccine Candidate VAR2CSA

**DOI:** 10.1371/journal.pone.0017942

**Published:** 2011-03-25

**Authors:** Vera V. Pinto, Sisse B. Ditlev, Kamilla E. Jensen, Mafalda Resende, Madeleine Dahlbäck, Gorm Andersen, Pernille Andersen, Thor G. Theander, Ali Salanti, Morten A. Nielsen

**Affiliations:** 1 Centre for Medical Parasitology at Department of International Health, Immunology and Microbiology, University of Copenhagen, Copenhagen, Denmark; 2 Department of Infectious Diseases, Copenhagen University Hospital (Rigshospitalet), Copenhagen, Denmark; Institut National de la Santé et de la Recherche Médicale - Institut Cochin, France

## Abstract

**Background:**

In *Plasmodium falciparum* malaria endemic areas placental malaria (PM) is an important complication of malaria. The recurrence of malaria in primigravidae women irrespective of acquired protection during childhood is caused by the interaction between the parasite-expressed VAR2CSA antigen and chondroitin sulfate A (CSA) in the placental intervillous space and lack of protective antibodies. PM impairs fetal development mainly by excessive inflammation processes. After infections during pregnancy women acquire immunity to PM conferred by antibodies against VAR2CSA. Ideally, a vaccine against PM will induce antibody-mediated immune responses that block the adhesion of infected erythrocytes (IE) in the placenta.

**Principal Findings:**

We have previously shown that antibodies raised in rat against individual domains of VAR2CSA can block IE binding to CSA. In this study we have immunized mice, rats and rabbits with each individual domain and the full-length protein corresponding to the FCR3 VAR2CSA variant. We found there is an inherently higher immunogenicity of C-terminal domains compared to N-terminally located domains. This was irrespective of whether antibodies were induced against single domains or the full-length protein. Species-specific antibody responses were also found, these were mainly directed against single domains and not the full-length VAR2CSA protein.

**Conclusions/Significance:**

Binding inhibitory antibodies appeared to be against conformational B-cell epitopes. Non-binding inhibitory antibodies reacted highly against the C-terminal end of the VAR2CSA molecule especially the highly polymorphic DBL6ε domain. Differential species-specific induction of antibody responses may allow for more direct analysis of functional versus non-functional B-cell epitopes.

## Introduction

Animal models are required for preclinical development of new generation vaccines against infectious diseases [1]–[3]. The ideal animal model mimics the human immunological response, the pathogen infection pathway, and allows analysis of the mechanism of the vaccine-induced protective immune response. However, despite the availability of humanised animal models such as transgenic mice [4], immunological responses in most animal models are only indicative of what to expect in humans. The development of a recombinant vaccine is initiated with identification and selection of an antigen that induces a desired immune response. At this step, multiple antigens are tested, which requires an inexpensive and easy to handle animal model. Following selection of antigen, the optimal route of vaccine administration together with a proper adjuvant formulation has to be evaluated. The design of the individual vaccine components and their delivery thus relies on the performance in these pre-clinical animal tests, emphasizing the importance of the animal model.

Vaccines are one of the future strategies to prevent and control malaria [5], one of the most widespread, pathogenic and deadly parasitic diseases in the world. Immunity is only acquired after successive infections in areas of high and stable malaria transmission [6]. Pregnant women become susceptible to placental malaria (PM) independent of any pre-existing immunity acquired during childhood. Placental malaria can have serious consequences for both mother and child such as maternal and infant anaemia, premature labour, low birth weight, and increased neonatal mortality [7]. However, after successive pregnancies women rapidly acquire immunity to PM, indicating that a vaccine strategy against PM may be feasible [8]. PM is caused by the binding of *Plasmodium falciparum* infected erythrocytes (IE) to chondroitin sulfate A (CSA) present on placental syncytiotrophoblast cells located in the intervillous space [9], [10]. Placental parasites express on the IE surface VAR2CSA, which is a *Plasmodium falciparum* Erythrocyte Membrane Protein 1 (PfEMP1) that mediates binding of the IE in the placenta [11], . VAR2CSA is a large (350 kDa) polymorphic protein with six Duffy-binding-like (DBL) domains, which complicates the development of a vaccine.

A potential animal model for studying protection induced by immunizations with recombinant domains of PfEMP1 could be chimpanzees (*Pan troglodytes*), whose natural malaria parasite, *Plasmodium reichenowi*, is closely related to *P. falciparum* [13]. However, for ethical and economic reasons this is not a feasible model for malaria vaccinology. To date, most malaria antigens are produced by recombinant technology and tested in lower mammalian species, such as rodents and rabbits. These models have allowed analysis of antibody responses to different combinations and boundaries of the six DBL-domains of VAR2CSA. Initially, we expressed all DBL-domains fromVAR2CSA-3D7 and found that all domains except DBL4ε, could induce antibodies against native VAR2CSA expressed on the surface of IE, with limited differences in immunizations of mice and rabbits [14]. However, these VAR2CSA-3D7 specific sera did not show significant inhibition of IE binding to CSA (unpublished data). Recently, in a large screening of the immunogenicity of different VAR2CSA antigens from the parasite line FCR3 using rat immunizations, we found that the DBL4ε domain of VAR2CSA could elicit the desired adhesion-inhibitory antibodies [15]. However, the induction of functional inhibitory antibodies to certain DBL-domains is poorly reproducible and the immune response tends to focus on non-adhesion blocking epitopes [16], [17]. In addition higher levels of inhibitory antibodies are acquired using full-length VAR2CSA (FV2) as compared to single DBL-domains [18]. To further investigate the induction of adhesion blocking antibody responses, all single recombinant domains of VAR2CSA-FCR3 were used in immunizations of mice, rats and rabbits, and compared to responses raised against the full length recombinant protein.

## Results

### Antigenicity of DBL-domains in three different animal species

**Figure 1:**
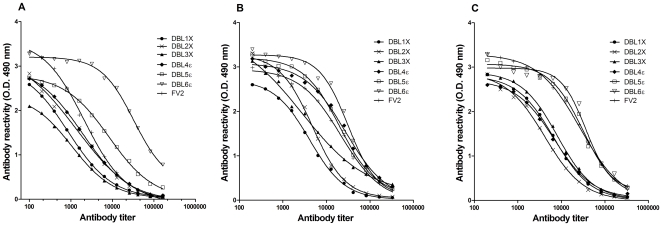
Antibody responses to recombinant proteins in different species of animals. The reactivity of VAR2CSA-specific IgG from mice (A), rats (B) and rabbits (C) immunized with individual domains (DBL1X-DBL6ε) and FV2 is shown as the mean of all animals immunized with a particular protein. Data are fitted to a sigmoid shaped dose response curve showing the different intrinsic antigenicity of the individual DBL-domains and FV2. For statistical analysis see Table S2.

To examine the antigenicity of the recombinant proteins the levels of antigen-specific IgG from immunized animals were measured using ELISA. All animal species immunized with individual DBL-domains or FV2 produced an IgG response, with a typical sigmoid shaped dose response curve of the antibody titrations (Figure 1). However, the levels of antibodies to each of the DBL-domains as well as to FV2 differed depending on the animal species used (for all statistical analysis of antigenic differences see Table S2). In mice, the DBL6ε immunizations resulted in the most potent IgG response with a significantly higher titre than that of FV2 and the other DBL-domains. Following DBL6ε in potency was DBL5ε and thereafter a lower response induced by DBL1X, DBL2X, DBL3X, DBL4ε and FV2 of similar potency. As observed in mice, immunizations of rats with DBL6ε resulted in a significantly higher IgG response compared to all other immunogens. DBL5ε and FV2 induced in rats a more potent response than DBL1X, DBL2X, and DBL3X (Figure 1B) but also DBL4ε induced a high antibody response. In rabbits, the seven tested antigens produced IgG responses that can be grouped in two according to the levels of responses (Figure 1C). The DBL6ε, DBL5ε and FV2 produced a high antibody response whereas IgG responses to DBL1X, DBL2X, DBL3X and DBL4ε domains were lower and essentially similar.

In summary, the tested VAR2CSA derived recombinant proteins induced medium to high levels of antibodies. FV2, DBL6ε and DBL5ε immunizations resulted in the highest antibody titers. FV2 appeared more antigenic in rabbits and rats compared to mice, and only rats appeared to induce high levels of antibodies to DBL4ε.

### Antibody reactivity against native VAR2CSA protein expressed on the surface of IE

Epitopes exposed in the recombinant single-domain proteins may not be surface exposed in the native full-length protein, primarily due to the proposed globular folding of the VAR2CSA molecule [19]. Therefore we tested the reactivity of the induced antibodies against native VAR2CSA exposed on the surface of the IE.

Erythrocytes infected with the FCR3 parasite line were used in flow-cytometry to test the IgG reactivity in animal sera against native VAR2CSA. The parasite cultures were continuously selected for binding to CSA by panning on BeWo cells, resulting in surface expression of VAR2CSA and sex specific recognition by IgG from human female serum, compared to male and Danish serum (data not shown).

**Figure 2:**
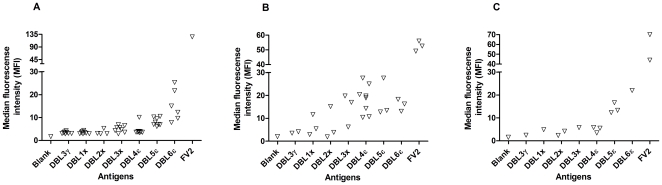
Antibody reactivity against native and surface expressed VAR2CSA. Serum from mice (A), rats (B) and rabbits (C) immunized with individual domains (DBL1X-DBL6ε) and FV2 was tested using FACS for reactivity against native VAR2CSA expressed on the surface of IE. Antibody surface reactivity is shown for FV2 and DBL1X-DBL6ε groups of animals as the median fluorescence intensity. Negative controls are serum from animals immunized with DBL3γVAR1 and a blank sample without serum.

In line with the ELISA results measuring the reactivity against the recombinant proteins, we found that the antibody reactivity against native VAR2CSA on the surface of the IE differed both with respect to animal species, and with antigen type. For mice and rabbits (Figure 2A and Figure 2C), the reactivity against native VAR2CSA on the IE surface was higher in sera from animals immunized with DBL5ε and DBL6ε, the reactivity against DBL1X, DBL2X, DBL3X and DBL4ε was low. In general, it appeared that rats acquired higher levels of antibodies specific to the native protein compared to mice and rabbits, the difference being especially marked for DBL4ε. However, as for mice and rabbits, the FV2, DBL5ε and DBL6ε immunizations resulted in high specific reactivity towards native VAR2CSA expressed on the IE (Figure 2B).

To test whether the reactivity against the native protein on the IE surface was associated with the apparent antigenicity of the recombinant domains, as measured by ELISA, the mean fluorescence values (MFI) was correlated to the EC50 titer values for all single domains. There was a highly significant linear correlation for mice and rabbits (data not shown) (r = 0.847, P<0.0001 & r = 0.809, P = 0.0008, respectively, Pearson), whereas the linear dependency for rats was somewhat lower (data not shown) (r = 0.476, P = 0.01, Pearson).

In summary, we found high levels of concordance between antibody reactivity against the recombinant domains and reactivity against the native protein in serum from rabbits and mice, but less so in serum from rats.

### Functional characteristics of antibodies against VAR2CSA domains

The proposed mechanism of protection against PM is that antibodies block adhesion of IE to CSA in the placenta [8]. Therefore, the functional capacity of the induced antibodies was measured in inhibition of CSA-binding assays. IE were incubated on decorin, either with or without specific serum or purified IgG. The specific inhibition was compared to the binding of IE incubated with negative control samples. Only sera from rabbits and rats were attained in sufficient volumes allowing three independent CSA binding experiments.

**Figure 3:**
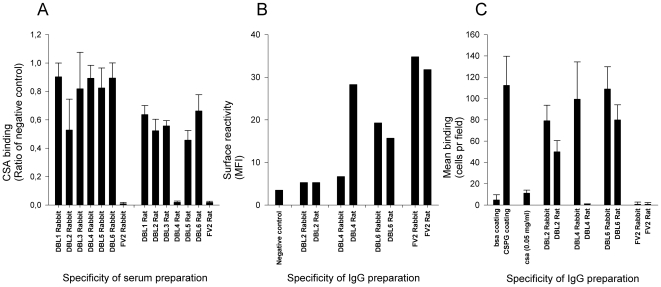
Comparison of surface reactivity and inhibition of FCR3-IE adhesion to CSPG. (A) Anti-VAR2CSA serum from rats and rabbits raised against DBL1X-DBL6ε and FV2 were tested using FCR3-IE for the ability of serum used in a 1∶10 dilution to inhibit binding to CSA in a 96-well plate assay. The data shown are the ratios of binding compared to the negative DBL3γVAR1 control ((Binding [test sample])/(Binding [negative control])). (B) IgG was purified from selected inhibitory and non-inhibitory sera and tested for reactivity in FACS at 1 mg/ml. DBL3γVAR1 was used as negative control. (C) Inhibition of binding to CSA using purified IgG at 0.5 mg/ml in the Petri dish assay. Error bars represents standard deviations.

The inhibitory capacity of the sera was tested prior to IgG purification, since crude purification of IgG may introduce a bias due to differential efficacy of purification of different antibody isotypes. Only FV2 and DBL2X specific rabbit serum appeared to inhibit more than the negative control rabbit serum (Figure 3A). This was in contrast to the responses measured using FCM against the native protein in DBL5ε and DBL6ε specific rabbit sera (Figure 2C), where high reactivity was found. The inhibitory capacity of rat serum was also not determined by the surface reactivity, for instance there was prominent inhibition using DBL4ε and FV2 specific serum and lack of inhibition by DBL6ε specific antibodies (Figure 3A), although DBL6ε specific antibodies also were highly reactive against native VAR2CSA (Figure 2B). To rule out that IgM or unspecific effects of serum did not mediate the inhibitory capacity, we purified IgG from both inhibitory and non-inhibitory sera and tested them in inhibition of binding assays at 0.5 mg/ml. The level of inhibition was essentially sustained in the IgG preparations (Figure 3B&C).

In summary, differential induction of functional antibody responses against DBL4ε, but not to the FV2 protein, were demonstrated in rats compared to rabbits.

### Anti-FV2 IgG reactivity against individual DBL-domains

To examine how the immune response against the full-length recombinant protein is focused with respect to individual DBL domains, we tested the levels of antibodies against individual DBL-domains in serum from animals immunized with FV2.

**Figure 4:**
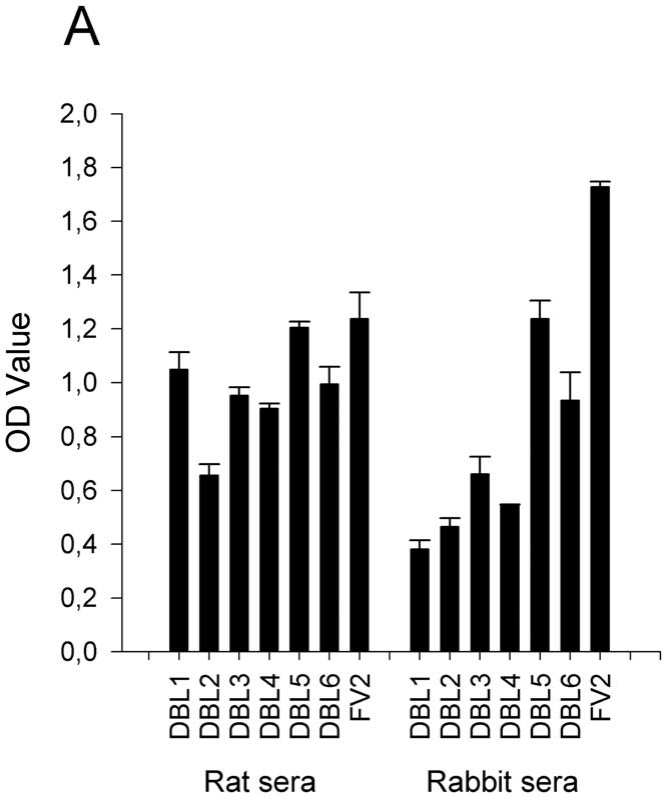
Reactivity of FV2-specific antibodies to single recombinant DBL-domains. The data shown are pools of serum from rats and rabbits immunized with FV2 tested in ELISA for reactivity towards single recombinant domains of VAR2CSA and FV2. Error bars represent standard deviations of triplicate measurements.

In general, we found that the pattern of antigenicity when immunizing with FV2 was similar to immunizations with single domains (Figure 4). In rats, FV2 antibodies targeted all DBL-domains with similar reactivity, with exception of DBL5ε and DBL2x, which showed highest and lowest reactivity, respectively. The levels of reactivity were more or less in line with the reactivity against the native protein of single-domain specific antibodies (Figure 2B). In rabbits, the reactivity against the domains appeared to be overall lower than for rats. Furthermore, the rabbit-FV2 reactivity was more focused towards DBL5ε and DBL6ε, as was also the case for the reactivity of single-domain specific antibodies towards the native erythrocyte surface exposed protein (Figure 2C).

In summary, it appeared that the pattern of antigenicity found when immunizing with single domains was essentially similar to immunizations with the FV2 protein in the different animals.

### Differential recognition of DBL4ε in ELISA-peptide array analysis

The DBL4ε recombinant protein appeared previously to be the only single domain that induces highly inhibitory antibodies [15]. However, the inhibitory antibodies were not induced in rabbits. Therefore, we tested the specificity of the antibody response in rabbits and rats against linear epitopes using a DBL4ε peptide ELISA.

**Figure 5:**
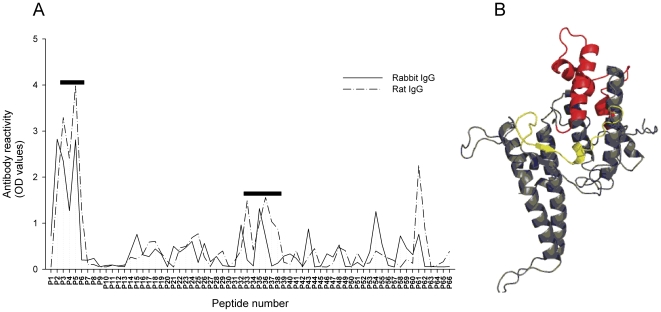
Antibody reactivity against DBL4ε epitopes measured by peptide analysis. (A) Mean IgG reactivity against DBL4ε peptides in individual serum samples from rats (n = 10) (Dash dot line) and rabbits (n = 4) (Solid line) immunized with DBL4ε recombinant protein is measured using ELISA. Black bars represent epitopes mapped on the structure model of DBL4ε. (B) The structure model of the DBL4ε domain. The major peak (p3–p6) is yellow and the minor peak (p33–p38) is red.

IgG induced by DBL4ε immunizations reacted with the DBL4ε peptides and no reactivity was detected with IgG from control IgG (data not shown). The overall level of reactivity was lower in rabbit serum (n = 4) compared to rat serum (n = 10) and a lower proportion of the rabbits reacted with a given peptide (data not shown). In general, the same areas of the linear sequence were targeted by IgG from the rats and rabbits, with the most distinct reactivity in both species of animals observed against peptide 2 to 5 (Figure 5A). However, it appeared that the rat antibody reactivity was skewed one to three peptides N-terminally compared to rabbit antibodies. Hence, the major peaks of reactivity recognized by rabbit-antibodies were peptides: 1–5, 14–19, 21–28, 31–33, 35–36 & 58–61, whereas rats recognized peptides 2–6, 14–20, 22–26, 31–38 & 60–63. Other peptides in the array were recognized by one species only, albeit some of them with quite low OD values. The major peak of reactivity (peptides 3–6) recognized by both rabbit-antibodies and rat-antibodies and one minor peak with distinct skewed reactivity (peptides 33–38) were mapped on the structure model (Figure 5B). Both these areas of the model were predicted to contain unstructured loops. In addition, peptide 3–6 contained a β-sheet hairpin motif and peptide 33–38 contained several α-helixes. The other minor peak primarily targeted by rat antibodies (peptides 61–62) was also characterized by unstructured amino acid sequences (data not shown).

In summary, the average reactivity to linear peptides covering the amino acid sequence of DBL4ε was, apparently slightly skewed, but otherwise remarkably similar between rabbits and rats.

## Discussion

The VAR2CSA protein is a vaccine candidate against PM due to its surface localisation, function as an adhesin for placental CSA and its role in immunity against PM [11], [20]–[27]. Although the full-length protein can be produced, a vaccine based on a single domain is more likely to be feasible, due to the complexity and size of the antigen. To analyze if the immune responses towards single-domains and the full-length protein were comparable, we tested all single VAR2CSA domains as well as the full-length protein in three different animal species.

In general, there was a direct association between antigenicity and reactivity to the surface of the infected erythrocyte. The reactivity against native VAR2CSA on the IE surface was higher in sera from animals immunized with DBL5ε, DBL6ε and FV2 compared to other domains (Figure 2), which was similar to the observed reactivity against the recombinant domains measured by ELISA (Figure 1). The correlations between the antigenicity of the domains and reactivity to the native protein on the surface of IE were highly significant in immunization of mice and rabbits, whereas the levels of rat antibodies reacting with the native protein in general were higher, which resulted in a less significant correlation. However, taken together the reactivity increased towards the C-terminal end of the external part of VAR2CSA, both in immunizations with single domains and FV2. This may reflect both an inherent antigenicity of single DBL domains of VAR2CSA and/or the accessibility of single domains in the quaternary structure of the native VAR2CSA molecule. This is in line with data showing that the functional CSA binding region is located N-terminally allowing for non-blocking antibodies to react with the C-terminal located domains (Dahlbäck et al submitted).

The inhibitory capacities of the induced rat and rabbit antibodies following FV2 immunizations were very high. However, the different species-specific inhibitory FV2 antibodies do not necessarily target the same epitopes, but further analysis of the epitope targets of FV2 specific antibodies are needed to elucidate the mechanism of inhibition. For immunizations with single domains, the inhibition of IE binding to CSA varied between animal species. Only rats immunized with DBL4ε induced IgG, which efficiently inhibited IE binding, whereas the rabbit sera specific against DBL4ε were non-inhibitory. The inhibitory effect of the IgG was not an effect of antibody titer, since immunizations with DBL5ε and DBL6ε also induced high titers, but these were in general non-inhibitory. These findings are in contrast to what others have found [21], [28]. Differences in observations could be due to different domain boundaries or to the variance in posttranslational protein modifications introduced for example by the baculovirus transfected *T. ni* as opposed to the *Pichia pastoris* expression system, such as differences in glycosylation pattern.

The above findings are not likely to be caused by differences in the MHC repertoire of the rat and rabbit strain used, since both strains used were outbred, reducing the likelihood that all of the individual rats and none of the rabbits induced functional antibodies. In addition, fair levels of antibodies was induced against the recombinant form of DBL4ε in rabbits, indicating appropriate T-cell responses during the immunizations, yet the DBL4ε specific rabbit sera was non-inhibitory even at rather high concentrations. Differential induction of binding-inhibitory antibodies is probably caused by species-specific differences in immuno-dominance of B-cell epitopes in different species of animals, similar to observations with antigens from other organisms [29], [30]. Responses to FV2 appeared more homogenous between species, at least with respect to the adhesion blocking properties, possibly due to presentation of more epitopes in a native form compared to single domains. Since the VAR2CSA molecule appears partly globular, immuno-dominant B-cell epitopes, not present in the native protein, may be exposed in individual DBL domains. This could also be the cause of the finding that antibodies induced against the DBL1X and DBL3X single domains, when based on heterologous sequences, can inhibit the binding of FCR3-IE [17], as opposed to antibodies induced against DBL1X and DBL3X, based on the FCR3 sequence [15].

Attempting to identify epitopes targeted by inhibitory antibodies, we compared differences in rabbit and rat antibody reactivity to linear peptides covering the entire DBL4ε domain. We previously did this with DBL4ε-FCR3, DBL4ε-3D7, DBL1X-3D7 and DBL3X-HB3 specific sera from rats [17]. The average reactivity to DBL4ε linear epitopes was apparently slightly skewed when comparing rat and rabbit antibodies. Otherwise, the majority of responses in rats and rabbits were surprisingly similar. This similarity, combined with the lack of functional activity of the rabbit antibodies, both with regard to surface activity and inhibitory capacity, probably shows that surface-reactive and binding-inhibitory rat antibodies are not targeting linear epitopes in the native protein. The most pronounced areas of reactivity against the linear sequence were mapped to regions in the DBL4ε model containing unstructured loops. This is in line with the findings that patches of high sequence diversity in VAR2CSA are concentrated in flexible loops [20], [31], [32] and that naturally acquired antibodies also are directed against these regions [33]. However, partly denatured antigen induced by Freund's adjuvant could increase the response to flexible loop regions in experimental immunizations [34]. Interestingly, a fraction of the expressed PfEMP1 during the maturation of the parasite in the IE remains intracellular [35]. Whether this intracellular pool of protein is denatured at the point of schizont rupture, promoting responses in natural infections to flexible loops more tolerant to mutations, as seen in other organisms [36], remains to be investigated.

It should be noted that DBL4ε and DBL4ε-ID4 specific antibodies are cross inhibitory on a panel of maternal parasite isolates [37], showing that a conserved region of this recombinant protein can be targeted by antibodies interfering with the interaction between VAR2CSA and CSA. We have not analyzed the cross-reactivity of the FV2 induced IgG, consequently it is unknown if the inhibitory response is directed towards conserved or variable regions. It is possible that selection pressure can increase exposure of immuno-dominant variable epitopes targeted by non-inhibitory antibodies.

In summary, it appears that the immunogenicity of the VAR2CSA protein is highest in the C terminal end, both immunizing with single domains and with the full-length protein. Identification of the epitopes targeted both by FV2 and DBL4ε specific binding inhibitory antibodies may serve as a tool to generate second-generation antigens by removing or masking redundant/unwanted epitopes. We do not know how the immune response will be focused in humans upon immunization with DBL4ε and the results from a phase 1 clinical trial in human volunteers with DBL4ε will be decisive for further clinical development. Retrospectively, we would probably not have identified the binding inhibitory capacity of DBL4ε specific antibodies had we only tested the antigen in rabbits and mice. This emphasises the value of testing different animal species as vaccination models, specifically for antigens like PfEMP-1 and EBA, which have evolved to generate variations in humoral immune responses.

## Materials and Methods

### Ethics statement

All procedures regarding animal immunizations complied with European and National regulations. This study was approved by the Danish animal welfare council under the Danish Ministry of Justice Approval ID: 2008/561-1498.

### Plasmodium falciparum culture

*P. falciparum*, FCR3 parasite (laboratory strain) were cultured in RPMI–1640 supplemented with 25 mmol/L sodium bicarbonate (Sigma–Aldrich), 0.125 µg/ml gentamycin, 0.125 µg/ml Albumax II (Invitrogen), 2% normal human serum and with 5% hematocrit of group 0+ human blood. To select for VAR2CSA expression, IE were repeatedly panned on BeWo-cells as described [38]. All isolates were mycoplasma negative and were regularly genotyped using nested GLURP and MSP-2 primers in a single PCR step.

### Protein production

All VAR2CSA single domains were cloned from genomic *P. falciparum* FCR3 parasite DNA (GenBank accession no. AY372123). Full length *var2csa* was based on a codon optimized synthetic DNA fragment as described [18]. Gene fragments were cloned into the *Baculovirus* vector, pAcGP67-A (BD Biosciences) modified to contain a V5 epitope upstream of a histidine tag in the C-terminal end of the constructs. Linearized Bakpak6 baculovirus DNA (BD Biosciences) was co-transfected with pAcGP67-A into Sf9 insect cells for generation of recombinant virus particles. Histidine-tagged recombinant protein was purified on Ni^2+^ sepharose columns from the supernatant of baculovirus infected High-Five insect cells using an ÄKTA-express purification system (GE-Healthcare). The full-length protein VAR2CSA (FV2) and the six VAR2CSA DBL-domains were produced corresponding to the following amino acid number in VAR2CSA: FV2FCR3: 1–2649, DBL1X: 58–438 (380 aa), DBL2X: 538–893 (355 aa), DBL3X: 1210–1587 (377 aa), DBL4ε: 1583–1947 (364 aa), DBL5ε: 1990–2328 (338 aa) and DBL6ε: 2307–2641 (334 aa).

### Animal immunizations

The animals were subcutaneously immunized with either single VAR2CSA-domain constructs (DBL1x-6ε) or with FV2. Control groups were immunized with the DBL3γ-VAR1-FCR3 in the same concentration as the tested VAR2CSA proteins [15]. Groups of BALB/c mice (n = 8) (inbred) (Taconic, Denmark) were subcutaneously immunized with 15 µg per animal of recombinant protein in Freund's complete adjuvant, followed by three follow-up immunizations of 10 µg of protein in Freund's incomplete adjuvant at two-week intervals. However, due to insufficient amounts of protein only four mice received DBL2x, and six mice received DBL6x. For immunizations with FV2 (done on a later time point) the mouse strain used was CB6F1 mice (F1 hybrid between BALB/c females and C57BL/6 males) (Harlan, USA) following the same protocol as previously described for the BALB-c mice. Seven groups of Wistar rats (outbred) (Taconic, Denmark) were immunized with 40 µg per animal of recombinant protein in Freund's complete adjuvant, followed by three booster injections of 20 µg of protein in Freund's incomplete adjuvant at 2½-week intervals. The rat groups consisted of three animals except the group receiving the DBL4ε (n = 8) and DBL3γ (n = 2). New Zealand White rabbits (outbred)(HB Lidköping Kaninfarm, Sweden) were immunized with 40 µg of recombinant protein in Freund's complete adjuvant, followed by three booster immunizations of 20 µg of protein in Freund's incomplete adjuvant at 3 week intervals. For each recombinant protein one rabbit was immunized with the exception of rabbits receiving DBL2x & FV2 (n = 2) and DBL4ε & DBL5ε (n = 3). For all animals sera were collected 8 days after the final immunization. Sera were stored at −20°C until use.

### ELISA

ELISA plates (Nunc Immunoplate) were coated with protein overnight (4°C) at a concentration of 1 µg/ml in 1× PBS. The plates were blocked with ELISA dilution buffer (1% BSA and 1% Triton x-100) before the serial diluted serum was added to the plate. Secondary horseradish peroxidase-conjugated (HRP) antibodies added in the following dilutions: anti-rabbit-IgG 1∶2000 (P0448, Dako), anti-mouse-IgG 1∶2000 (P0260, Dako), and anti-rat-IgG 1∶3000 (A9037, Sigma). The optical density was measured at 490 nm in an ELISA plate reader (VersaMax, Molecular Devices). Antibody titration curves were determined using GraphPad Prisma5.

### Affinity purified FV2 specific antibodies

The FV2-specific IgG from both rats and rabbit's sera were affinity purified on HiTRap™ NHS-activated HP columns (GE Healthcare), according to the manufacturer's instructions. Briefly, each column was coated with 0.5 mg/ml of a DBL-domain resulting in six different columns for the individual six DBL-domains. The affinity purified IgG was verified using ELISA as previously described in this material and methods section. The assay was performed twice with similar results.

### Flow-cytometry

The reactivity of IgG in animal sera to the VAR2CSA protein expressed on IE surface was measured by flow-cytometry (FCM) using a protocol modified from [39]. Briefly, the parasite culture was enriched for late trophozoite and schizont stage parasites in a strong magnetic field (MACS, Miltenyi). Aliquots of 2×10^5^ IE in a total volume of 100 µl were labelled by ethidium bromide and sequentially exposed to (1) 15 µl animal serum and (2) 1∶100 dilutions of FITC labelled secondary antibodies specific for IgG from the individual species: Mouse (FL2000, Vector); rat (62–9511, Invitrogen) and rabbit (FL1000, Vector). As a negative control, IE were incubated both with sera from non-immunized animals and without animal serum and then exposed to secondary antibodies specific against IgG from different animals spec. Data from 5000 infected cells (ethidium bromide positive) was collected on a FC500 flowcytometer (Beckman Coulter). The median FITC fluorescence intensity was determined using Winlist Software (Verity Software House).

### Inhibition of CSA-binding assay

For analyses of the capacity of serum to inhibit binding we used 2×10^5^ tritium-labeled late-stage IE and 15 µl serum in a total volume of 120 µl which were added in triplicates to wells coated with 2 µg/ml of the commercially available chondroitin sulfate proteoglycan Decorin (D8428; Sigma-Aldrich). Decorin was used as a source of CSA as this has a protein core resulting in more efficient coating to plastic than CSA. After incubation for 90 min at 37°C, unbound IE were washed away by resuspension performed by a pipetting robot (Beckman Coulter). The proportion of adhering IE was determined by liquid scintillation counting on a Topcount NXT (Perkin-Elmer).

The inhibitory capacity of the induced VAR2CSA domain-specific IgG was measured using a standardized Petri dish method both due to the smaller volume in this assay, and because this a more commonly used assay [40]–[42]. IgG was purified from the animal sera using HiTrap Protein G HP kit (GE Healthcare) and dialyzed against PBS 1× buffer. Briefly, 20 spots in each Petri dish (Falcon 351005) were coated overnight with 20 µl of decorin (2 µg/ml in PBS). Spots were blocked with 3% bovine serum albumin. Parasite cultures were enriched for late trophozoite and schizont stages in a strong magnetic field (MACS, Miltenyi) and adjusted to reach a 20% parasitemia at 0.5% hematocrit. The parasite suspension was pre-incubated with both animal sera and purified IgG at different concentrations at 37°C for 30 min. Duplicate spots were incubated for 15 min at 37°C in a humid chamber. Unbound erythrocytes were removed by washing with PBS on a gyro-turntable (Stuart) until spots were clear of blood. Bound erythrocytes were glutaraldehyde-fixed (1.5%, 5 min) and Giemsa stained. Five fields were counted in each spot using a magnification of 20× using a Leica Microscope. As negative controls, anti-DBL3γVAR1 serum and IgG were used. Parasite binding to decorin was abrogated by soluble CSA (Sigma-Aldrich) and by chondroitinase (Sigma-Aldrich) treatment of decorin (data not shown).

### Peptide analysis

Epitope mapping was done using a peptide array covering the DBL4ε domain. The peptide array consisted of 66 synthetic 16–28 amino acids long overlapping peptides, spanning amino acids 1607–1989 of the *P. falciparum*-FCR3 *var2csa* sequence (Table S1). The peptides were prepared by Schafer-N Copenhagen and the purity of the peptides was expected to be 70% or higher. Peptide binding was determined using ELISA. ELISA plates (Nunc Immunoplate) were coated overnight (4°C) with DBL4ε peptides at a concentration of 3 µg/ml in 1× PBS. Plates were blocked with ELISA dilution buffer (1% BSA and 1% Triton x-100) before animal serum samples were added. The serum samples were diluted 1∶100. The following secondary horseradish peroxidase-conjugated (HRP) antibodies were used: anti-rabbit P0448 (DAKO), dilution 1∶2000; and anti-rat A9037 (Invitrogen) and diluted 1∶3000. The optical density was measured at 490 nm in an ELISA plate reader (VersaMax, Molecular Devices). The level of reactivity in this ELISA based peptide array was directly associated to the level of reactivity in an assay where identical peptides were synthesised directly on to a chip, confirming homogeneous coating of the peptides [17].

### Structure modelling

The structure model of the DBL4ε sequence (C1576–C1910) was created using the same method as described for other VAR2CSA DBL-domains [20], but using as a template the newly published structure of DBL3X-VAR2CSA (3BQK) [43].

### Statistical analyses

Best-fit curves and mean EC50 values to determine compare antibody titres were done using Gradhpad Prism5 (Gradhpad Software Inc.). Sigma Stat 3.11 (Sysstat software Inc.) was used in the following analysis: one way ANOVA tests followed by multiple pair wise comparison procedures (Holm-Sidak method), which were used to determine differences between Log EC50 values to measure the antigenicity of single domains and FV2; Pearson correlation test, which was used to assess the correlation between MFI values and OD-EC50 values.

## Supporting Information

Table S1 Position and sequence of amino acids in peptides used in the ELISA peptide array.
(DOC)

Table S2 Statistical analysis of the pattern of reactivity in ELISA to single DBL-domains and FV2 performed for each species of animal separately.
(DOC)
