# Supplementary material for: Differential Induction of Functional IgG Using the Plasmodium falciparum Placental Malaria Vaccine Candidate VAR2CSA
Source: PLoS One. 2011 Mar 25;6(3):e17942. doi: 10.1371/journal.pone.0017942 (PMC3064590; doi:10.1371/journal.pone.0017942)
Supplement: Table S1 — Position and sequence of amino acids in peptides used in the ELISA peptide array. (DOC) [file pone.0017942.s001.doc]

Table S1. Position and sequence of amino acids in peptides used in the ELISA peptide array

| **No.** | **Position** | **Amino Acid sequence** | **No.** | **Position** | **Amino Acid sequence** |
| --- | --- | --- | --- | --- | --- |
| 1 | 1607-1622 | KKYIKKLENGRSLEGV | 34 | 1717-1732 | DSKLNEIFGSSNTNDI |
| 2 | 1610-1625 | IKKLENGRSLEGVYVP | 35 | 1725-1740 | GSSNTNDIDTKRARTD |
| 3 | 1613-1628 | LENGRSLEGVYVPPRR | 36 | 1733-1748 | DTKRARTDWWENETIT |
| 4 | 1616-1631 | GRSLEGVYVPPRRQQL | 37 | 1741-1756 | WWENETITNGTDRKTI |
| 5 | 1619-1634 | LEGVYVPPRRQQLCLY | 38 | 1749-1764 | NGTDRKTIRQLVWDAM |
| 6 | 1622-1637 | VYVPPRRQQLCLYELF | 39 | 1757-1772 | RQLVWDAMQSGVRYAV |
| 7 | 1625-1640 | PPRRQQLCLYELFPII | 40 | 1765-1780 | QSGVRYAVEEKNENFP |
| 8 | 1628-1643 | RQQLCLYELFPIIIKN | 41 | 1773-1788 | EEKNENFPLCMGVEHI |
| 9 | 1631-1646 | LCLYELFPIIIKNEEG | 42 | 1781-1796 | LCMGVEHIGIAKPQFI |
| 10 | 1634-1649 | YELFPIIIKNEEGMEK | 43 | 1789-1804 | GIAKPQFIRWLEEWTN |
| 11 | 1637-1652 | FPIIIKNEEGMEKAKE | 44 | 1797-1812 | RWLEEWTNEFCEKYTK |
| 12 | 1640-1655 | IIKNEEGMEKAKEELL | 45 | 1805-1820 | EFCEKYTKYFEDMKSK |
| 13 | 1643-1658 | NEEGMEKAKEELLETL | 46 | 1813-1828 | YFEDMKSKCDPPKRAD |
| 14 | 1646-1661 | GMEKAKEELLETLQIV | 47 | 1821-1836 | CDPPKRADTCGDNSNI |
| 15 | 1649-1664 | KAKEELLETLQIVAER | 48 | 1829-1844 | TCGDNSNIECKKACAN |
| 16 | 1652-1667 | EELLETLQIVAEREAY | 49 | 1837-1852 | ECKKACANYTNWLNPK |
| 17 | 1655-1670 | LETLQIVAEREAYYLW | 50 | 1845-1860 | YTNWLNPKRIEWNGMS |
| 18 | 1658-1673 | LQIVAEREAYYLWKQY | 51 | 1854-1868 | RIEWNGMSNYYNKIYR |
| 19 | 1661-1676 | VAEREAYYLWKQYNP | 52 | 1861-1876 | NYYNKIYRKSNKESED |
| 20 | 1664-1679 | REAYYLWKQYNPTGKG | 53 | 1869-1884 | KSNKESEDGKDYSMIM |
| 21 | 1667-1682 | YYLWKQYNPTGKGIDD | 54 | 1877-1892 | GKDYSMIMAPTVIDYL |
| 22 | 1670-1685 | WKQYNPTGKGIDDANK | 55 | 1885-1900 | APTVIDYLNKRCHGEI |
| 23 | 1673-1688 | YNPTGKGIDDANKKAC | 56 | 1893-1908 | NKRCHGEINGNYICCS |
| 24 | 1676-1691 | TGKGIDDANKKACCAI | 57 | 1901-1916 | NGNYICCSCKNIGAYN |
| 25 | 1679-1694 | GIDDANKKACCAIRGS | 58 | 1909-1924 | CKNIGAYNTTSGTVNK |
| 26 | 1682-1697 | DANKKACCAIRGSFYD | 59 | 1917-1932 | TTSGTVNKKLQKKETE |
| 27 | 1685-1700 | KKACCAIRGSFYDLED | 60 | 1925-1940 | KLQKKETECEEEKGPL |
| 28 | 1688-1703 | CCAIRGSFYDLEDIIK | 61 | 1933-1948 | CEEEKGPLDLMNEVLN |
| 29 | 1691-1706 | IRGSFYDLEDIIKGND | 62 | 1941-1956 | DLMNEVLNKMDKKYSA |
| 30 | 1694-1709 | SFYDLEDIIKGNDLVH | 63 | 1949-1964 | KMDKKYSAHKMKCTEV |
| 31 | 1697-1712 | DLEDIIKGNDLVHDEY | 64 | 1957-1972 | HKMKCTEVYLEHVEEQ |
| 32 | 1700-1717 | DIIKGNDLVHDEYTKYI | 65 | 1965-1980 | YLEHVEEQLNEIDNAI |
| 33 | 1709-1724 | HDEYTKYIDSKLNEIF | 66 | 1973-1989 | LNEIDNAIKDYKLYPLD |
