# Supplementary material for: Differential Induction of Functional IgG Using the Plasmodium falciparum Placental Malaria Vaccine Candidate VAR2CSA
Source: PLoS One. 2011 Mar 25;6(3):e17942. doi: 10.1371/journal.pone.0017942 (PMC3064590; doi:10.1371/journal.pone.0017942)
Supplement: Table S2 — Statistical analysis of the pattern of reactivity in ELISA to single DBL-domains and FV2 performed for each species of animal separately. (DOC) [file pone.0017942.s002.doc]

**Table S2** Statistical analysis of the pattern of reactivity in ELISA to single DBL-domains and FV2 performed for each species of animal separately

| Domain | Mice  EC 50  95% C.I. | Significantly higher than:  (P value) | Rat  EC50  95% C.I. | Significantly higher than:  (P value) | Rabbit  EC50  95% C.I. | Significantly higher than:  (P value) |
| --- | --- | --- | --- | --- | --- | --- |
| DBL1X | [447.9-1663] | None | [3053-5547] | DBL3  (P = 0.007 ) | [5721-7991] | None |
| DBL2X | [128.1-14487] | None | [3663-6227] | DBL3  (P = 0.006) | [3203-5428] | None |
| DBL3X | [774.6-1481] | None | [1777-4263] | None | [7631-8524] | None |
| DBL4ε | [1192-2949] | DBL1  (P = 0.005) | [19874-38217] | DBL1, 2, 3 & 5  (P < 0.009) | [4766-9872] | None |
| DBL5ε | [5860-11912] | DBL1, 3, 4 & FV2  (P < 0.0001 ) | [16046-25561] | DBL1, 2 & 3  (P < 0.005) | [14276-71291] | DBL1, 2, 3 & 4  (P < 0.004) |
| DBL6ε | [20649-52816] | All other domains  (P < 0.0001) | [23201-42949] | DBL1, 2, 3, 5 & FV2  (P < 0.006) | [29178-45383] | DBL1, 2, 3 & 4  (P < 0.004) |
| FV2 | [1416-3135] | None | [16497-28095] | DBL1, 2 & 3  (P < 0.005) | [9464-77160] | DBL1, 2, 3 & 4  (P < 0.005) |
